# The Hardier You Are, the Healthier You Become. May Hardiness and Engagement Explain the Relationship Between Leadership and Employees’ Health?

**DOI:** 10.3389/fpsyg.2018.02784

**Published:** 2019-01-14

**Authors:** Greta Mazzetti, Michela Vignoli, Gerardo Petruzziello, Laura Palareti

**Affiliations:** ^1^Department of Education Studies, University of Bologna, Bologna, Italy; ^2^Department of Psychology and Cognitive Science, University of Trento, Rovereto, Italy; ^3^Department of Psychology, University of Bologna, Bologna, Italy

**Keywords:** transformational leadership, hardiness, work engagement, general health, Job Demands-Resources model

## Abstract

The main goal of this study was to delve deeper into the relationship between transformational leadership and better general health status among employees. Based on the Job Demands-Resources model of occupational well-being, the current research investigated the role of transformational leadership, as a job resource, in fostering individual hardiness, as a personal resource, which may in turn result in higher levels of work engagement and, consequently, better general health status among employees. Data were collected from 358 white-collar employees in an Italian company. Most of them were women (52.9%) with a mean age of 44.42 years (*SD* = 9.22). To evaluate the hypothesis of a mediating role of employees’ hardiness and work engagement within the relationship between transformational leadership and workers’ general health, a bootstrapping approach was tested using a serial mediation model. In the current sample, enhanced levels of hardiness and work engagement among employees mediated the association between perceived levels of transformational leadership and individual general health conditions. These findings corroborated the role of transformational leadership as a strategic job resource in enhancing employees’ hardiness and engagement with their work, which may in turn protect their general health status. Organizations willing to rely on a healthy workforce should implement human resource management strategies focused on leadership training capable of boosting employees’ hardiness.

## Introduction

Nowadays the quality of employees’ motivation, performance, and functioning has become a crucial issue among scholars and practitioners focused on identifying interventions capable of improving employees’ working experience and organizational effectiveness. Positive organizational psychology can frame these attempts, since this theoretical perspective has become crucial in shaping strategies aimed at empowering employees and helping them benefit from their job activities and, at the same time, reach critical goals, rather than focusing on mere prevention of symptoms of disease and illness (Luthans, 2017). This approach could become a particularly effective way to recognize, evaluate, and cultivate employees’ human and social strengths, to assist them in expressing their potential, and enhance their performance and well-being (Meyers et al., 2013; Youssef-Morgan and Luthans, 2015). From this perspective, the specific approach applied to human resources management acting within organizations could considerably affect employees’ well-being and their attainment of crucial organizational outcomes. In particular, transformational leadership – a style of managing people in which leaders act to improve, inspire, and stimulate followers, aiming to help them grow – may foster employees’ well-being and, therefore, it may enable employees to fulfill their potential, make suitable decisions, and effectively perform their tasks, and thereby be a key to an organization’s success (Ng, 2017). Besides the large body of research that supports this direct relationship, the academic literature reports several attempts to explain the mechanism that links this leadership style to the level of well-being reported by employees (Arnold, 2017). Moreover, research on the potential role of hardiness – a personality composite of beliefs concerning oneself and the surrounding environment that stems from a combination of a sense of commitment, control, and challenge (Kobasa et al., 1982) – is still lacking that can explain this link.

This study draws on the idea that a transformational leader could, through his/her behaviors, shape hardiness components of his/her employees, providing them with a sense of confidence and control, encouraging them to commit to organizational goals, and strive to actively face stressful and challenging situations. Thus, drawing on the motivational process of the Job Demands-Resources model (JD-R model; Schaufeli and Taris, 2014), the current study aimed at delving more deeply into the relationship between transformational leadership, as a major organizational resource, and employees’ well-being, through subsequent mediation of followers’ hardy personality and work engagement. This study attempts to shed light on one way in which supervisors could make a contribution to promoting employees’ well-being and strengths, thus helping them to face challenging job demands, which could otherwise harm workers’ well-being (Bakker and Demerouti, 2017).

### Transformational Leadership

Bass (1985) proposed *transformational* leadership as a style of managing human resources, essentially based on behaviors focusing on connecting with, and empowering followers to achieving common organizational goals. According to the renowned definition put forward by Avolio and Bass (1995) and Avolio et al. (1999) transformational leadership results from the combination of four primary behaviors: (1) *Individual consideration* refers to a leader’s ability to acknowledge employees’ capabilities and needs, and to meet them with empathy and support, thus fostering trust and satisfaction; (2) *Inspirational motivation* refers to the definition and sharing of positive and meaningful goals with the aim of inspiring and challenging employees to achieve more than the expected results; (3) *Intellectual stimulation* involves stimulating employees to seek better ways to fulfill their tasks and coaching them in making their own decision, on the basis of their own ideas; (4) *Idealized influence* entails the leader’s tendency to act as a role model for his/her followers, encouraging them to identify with him/her and perform desirable behaviors, thus trying to develop new leaders. There is compelling evidence from transformational leadership research showing a strong relationship with several positive outcomes (both at individual and team levels), such as individual and group task performance and effectiveness (Gilmartin and D’Aunno, 2007; Hoffman et al., 2011), followers’ extra-role performance (Salanova et al., 2011), followers’ creativity (Wang and Rode, 2010), and psychological empowerment (Wang and Howell, 2012). On the other hand, research on the variables that may explain the relationship between transformational leadership and positive outcomes (i.e., employees’ well-being) is far from being exhausted.

As outlined by Nielsen and Munir (2009), transformational leadership may have a positive effect on employees’ health, considered both as the absence of illness conditions and, in a broader sense, as a general state of well-being, and thus comprising a multi-dimensional construct (including job and life satisfaction, general health, vitality, quality of one’s relationships, and environmental mastery). For instance, Kara et al. (2013) indicated that transformational leadership predicts higher levels of employees’ quality of life. In a similar vein, Jacobs et al. (2013) revealed that employees perceiving greater transformational leadership are more likely to experience greater psychological well-being. Moreover, employees exposed to transformational behaviors are more likely to experience a higher level of job satisfaction, as well as lower levels of emotional exhaustion, and of symptoms of stress and anxiety (Cummings et al., 2010; Skakon et al., 2010). Thus, transformational leadership represents a crucial organizational resource in promoting a healthy work environment. Accordingly, researchers have made several attempts to shed light on the psychological mechanism underlying the relationship between transformational leadership and positive outcomes expressing different facets of employees’ well-being. In this respect, Nielsen and Daniels (2016) showed that transformational leaders may shape employees’ perception of their working conditions, which in turn may affect well-being. More generally, transformational leaders may positively impact employees’ experience and well-being through the reinforcement of resources that are related to employees’ morale and health. Among them, a particular role has been attributed to individual resources.

Hence, variables that can mediate the relationship between transformational leadership and employees’ well-being include autonomous motivation (Fernet et al., 2015), general self-efficacy (Liu et al., 2010), and occupational self-efficacy (Perko et al., 2014). The aim of the current study is to contribute to the understanding of this relationship through the investigation of the mediating role that a further personal resource (i.e., hardiness) and the consequent motivational job-related state (i.e., work engagement) may play in explaining the impact of transformational leadership and employees’ well-being. In particular, the following hypothesis was formulated:

***Hypothesis 1.*** Transformational leadership (as a job resource) would be positively associated with greater levels of employee health.

### Employees’ Hardiness

Employees’ hardiness (also known as hardy personality) represents a personality structure originally described by Kobasa (1979) and entailing a committed and meaningful approach to life and life activities, a greater sense of control of life events, and the aptitude to experience changes as challenges and opportunities for growth and learning. In particular, this personality trait is defined as the combination of three underlying dimensions: (1) *commitment*, which entails the tendency to be constantly involved in daily life activities, as well as an inner interest in things and people pertaining to the surrounding environment; (2) *control*, which is defined as strong confidence in one’s ability to control and significantly influence life events; and (3) *challenge*, which involves the tendency to consider changes as natural and desirable events that may represent concrete opportunities for learning and personal growth (Maddi, 2008). These facets imply that hardiness may be considered as a personal resource that acts as a protective factor for employees facing demanding and threatening situations and promotes adaptive behaviors. Hardy individuals tend to reframe stressful situations and job demands, resulting in a more optimistic and challenging appraisal, thus adopting more effective coping strategies, focused on the problem rather than on an avoidant approach. Empirical findings have consistently revealed a negative association between hardiness and burnout symptoms, since this personal resource allows employees to perceive events as meaningful, challenging, and under control, thus preventing negative outcomes such as depersonalization and depletion of cognitive and emotional energy (Lo Bue et al., 2013; Adriaenssens et al., 2015).

Although hardiness has been conceptualized as a personal characteristic, this resource is relatively malleable; thus, it could be fostered by an organizational context conducive to its development (Sudom et al., 2014). As previously stated, previous results suggested that transformational leadership boosts employees’ self-efficacy, especially through the key dimensions of idealized influence and inspirational motivation (Nielsen and Munir, 2009). These facets play a strategic role principally in enhancing the control component of hardiness, since they foster employees’ confidence in their ability to efficiently influence their job and work environment. Furthermore, transformational leadership could also enhance the commitment dimension of hardiness. Through behaviors pertaining to the dimension of idealized influence, this leadership style prompts employees to proactively engage in carrying out organizational processes, thus experiencing a greater commitment to and identification with their work goals (Stone et al., 2004). Moreover, the challenge dimension of hardiness may be enhanced by inspirational motivation behaviors, since they induce employees to re-appraise stressful events as worthwhile, encouraging them to identify the positive side of threats. Additionally, intellectual stimulation may motivate employees to seek and shape creative solutions to tackle individual, group, and organizational setbacks, thus promoting the implementation of active coping strategies, while the dimension of individual consideration may drive employees to feel more valued and supported when facing stressful and demanding situations (Harland et al., 2005).

As emphasized by such empirical evidence and posited in the academic literature (Bartone, 2006), the current research hypothesized that transformational leadership may promote individuals’ sense of control, commitment, and challenge, thus showing a positive association with employees’ hardiness. Thus, the following hypothesis was tested:

***Hypothesis 2.*** Employee hardiness would mediate the association between transformational leadership and employees’ general health.

### Hardiness and Work Engagement

One of the prominent job stress models of the last 20 years is the JD-R model (Schaufeli and Taris, 2014), which postulates the presence of a motivational process by which job resources, defined as those psychological and organizational aspects that allow employees to achieve their goals and stimulate them to learn from and grow in their job, boost employees’ work engagement. Engagement is described as a positive work-related condition stemming from the combination of vigor (i.e., the willingness to invest greater energy at work and the individual persistence in face of difficulties), dedication (i.e., the feeling of passion, involvement, and challenge toward the job), and absorption (i.e., the inclination to stay extremely focused on, and immersed in, the job). A large number of literature reports have revealed that work engagement constitutes a main antecedent of positive organizational and individual outcomes, such as improved in-role and extra-role job performance (Christian et al., 2011), as well as employees’ well-being (Schaufeli, 2013).

The current study aimed at exploring the role of transformational leadership as a job resource that can foster employees’ engagement by playing the role postulated within the JD-R model, that is, the satisfaction of basic needs and the attainment of significant work goals. Namely, by acting with integrity as a role model, by providing inspiration, and showing concern for employees’ needs, and through awareness of their different qualities and ambitions, transformational leadership acts as a job resource that conveys an inspirational vision, encourages critical reasoning, and proposes creative ideas, and thus employees may be driven to uncover a renewed feeling of passion and energy toward their work (Gong, 2010). As a result, employees are more likely to feel emotionally, cognitively, and physically engaged in achieving their work goals.

Overall, these results suggest that employees of a transformational leader may experience a greater sense of coherence and satisfaction about their functioning, self-confidence, and exhibit greater motivation, indicating higher levels of well-being. This reasoning led us to develop the following research hypothesis:

***Hypothesis 3.*** The relationship between transformational leadership and general health would be mediated by the level of employees’ work engagement.

### Hardiness and Engagement

Empirical evidence based on the JD-R model indicates that job resources may indirectly enhance employees’ engagement through strengthening of personal resources, which describes those subjective characteristics that allow individuals to have control over their work environment and cope effectively with job demands (Mazzetti et al., 2016a). In addition to job resources, personal resources may not only boost employees’ work engagement, but also prevent the occurrence of burnout symptoms. In line with this definition, hardiness may represent a personal resource that can foster employees’ engagement with their jobs. Hardy people tend to be committed to, and to give value to, their work activities, continuing in their efforts even in the face of adversity. Besides the recognized role of hardiness in preventing burnout, empirical results have also supported the role of this individual characteristic as an antecedent of work engagement (Moreno-Jiménez et al., 2012; Lo Bue et al., 2013). In line with the empirical findings discussed, the current research hypothesized that work engagement mediates the relationship between employees’ hardiness and health status.

**FIGURE 1:**
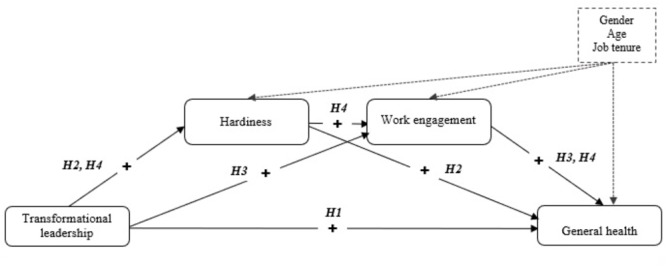
The hypothesized serial mediation model.

The aim of the current study was to elucidate potential mechanisms underlying the relationship between transformational leadership and employees’ health, through a serial mediation model that considers the role of employees’ hardiness as a personal resource that can foster employees’ work engagement and, consequently, employees’ health and well-being (Figure 1). Based on the existing related theory and summarized research, the following hypothesis was formulated:

***Hypothesis 4.*** Transformational leadership (as a job resource) would be associated with higher levels of employee health, through the subsequent mediation, first of employee hardiness (as a personal resource), and then of work engagement.

## Materials and Methods

### Participants and Procedures

The current study was part of a research project focused on occupational health and well-being among employees working in an Italian company that develops marketing and communication policies and manages the process of purchasing and distributing goods for one of the major Italian companies from the large-scale retail sector. In particular, employees participated in short presentation sessions held by two members of the University research group aimed at presenting the general aims of the project. The questionnaire included a cover letter that summarized the contents and goal of the study and emphasized participant anonymity and information confidentiality, in accordance with the guidelines for personal data treatment defined by the Italian privacy law (Law Decree DL-196/2003); thus, participants’ informed consent was implied through survey completion. The cover letter also stated that the employer would not be informed of any participants’ decision not to complete the survey. With regard to ethical standards for research, the study adhered to the latest version of the Declaration of Helsinki (World Medical Association, 2013); therefore, ethics approval was not compulsory, as per applicable institutional and national guidelines. Additional ethical approval was not required, since there was no treatment, including medical, invasive diagnostics, or procedures causing psychological or social discomfort for the participants. Researchers invited all employees to fill out the paper-and-pencil questionnaire.

A total of 358 white-collar workers from 456 eligible employees completed the questionnaire as a part of a psychosocial risk assessment project. Hence, the response rate of the present study was equal to 78.51%. Most participants were women (52%) and the mean age was 44.42 years (*SD* = 9.22). Concerning the specific work roles involved, participants were employed at the Sales and Buying unit (33.2%), the Production and Quality unit (22%), the Distribution/Logistics unit (21.1%), the Human Resources department (13.6%), and the Marketing Research and Advertising unit (10.1%). Most participants had a permanent employment contract (94.4%) and worked full-time (86.7%). Moreover, the mean job tenure in the current organization was 14.65 years (*SD* = 8.96).

### Measures

*Transformational leadership* was measured using the subscales assessing *individualized consideration, inspirational motivation*, and *idealized influence* contained in the Multifactor Leadership Questionnaire (MLQ; Bass and Avolio, 2000). Each subscale included four items, all scored on a Likert scale that ranges from 1 (*totally disagree*) to 5 (*totally agree*). A sample item is “My supervisor treats each of us as individuals with different needs, abilities, and aspirations.” Although an Italian validation does not exist, this scale has been used in previous studies with an Italian sample (Vignoli et al., 2018). Cronbach’s alpha was 0.93.

*Hardiness* was measured through the Italian version (Mazzetti et al., 2016b) of the Occupational Hardiness Questionnaire developed by Moreno-Jiménez et al. (2014). This survey includes three subscales of five items each: *challenge* (e.g., “When possible I look for new and different situations in my work environment”), *control* (e.g., “I do everything I can to make sure I control the results of my work”), and *commitment* (e.g., “My daily work satisfies me and makes me totally dedicated to it”). All items were rated on a four-item Likert scale from 1 (*completely disagree*) to 4 (*completely agree*). Cronbach’s alpha was 0.77.

*Work engagement* was rated using the Italian validation (Balducci et al., 2010) of the short version of the Utrecht Work Engagement Scale (UWES-9; Schaufeli et al., 2006) consisting of three subscales reflecting the core dimensions of the construct: *vigor* (e.g., “At my work, I feel I’m bursting with energy”), *dedication* (e.g., “I am enthusiastic about my job”), and *absorption* (e.g., “I feel happy when I am working intensely”). Each subscale consisted of three items scored on a seven-point frequency scale that ranged from 0 (*never*) to 6 (*always*). Cronbach’s alpha was 0.90.

*General health* was measured using the brief Italian version (Fraccaroli and Schadee, 1993) of the General Health Questionnaire (GHQ-12; Goldberg, 1978), a widely used measure assessing the absence of minor psychiatric symptoms. The scale consists of 12 items covering two main dimensions: *general dysphoria* (mainly referring to anxiety and depressive symptoms) and *social dysfunction* (concerning the accomplishment of daily activities and self-evaluated coping ability). Using a four-point frequency scale, participants answer questions about how they have been feeling over the previous 4 weeks, with higher scores indicating better health (e.g., “Over the past few weeks, have you been able to face up to your problems?”). Cronbach’s alpha was 0.85.

### Analysis Strategy

The research hypothesis was tested using a bootstrapping approach as outlined by Hayes (2018). This method is appropriate when sample sizes are relatively small because it produces a distribution using the observed data, from which statistical effects are estimated (Preacher and Hayes, 2008). In particular, the mediation model with multiple mediators operating in serial implemented in the SPSS macro PROCESS (model 6) allowed to assess the current model. To derive the direct and indirect effects, the model estimated all path coefficients simultaneously. Moreover, the bootstrapping method generated an estimate of the indirect effect, including a 95% confidence interval. When zero is not included in the 95% confidence interval, one can conclude that the indirect effect of the independent variable (i.e., transformational leadership) on the dependent variable (i.e., general health) is mediated by the proposed sequential mediators (i.e., hardiness and work engagement). Based on previous empirical results, we decided to control for the potential confounding effect of participants’ gender, age, and job tenure (Garrosa et al., 2008; Harju et al., 2016; Oskrochi et al., 2018).

## Results

### Descriptive Results

Table 1 reports the correlations among the study variables. As expected, transformational leadership reported a positive relationship with hardiness, work engagement and general health. Furthermore, the proposed mediators (i.e., hardiness and work engagement) were positively associated to each other as well as with the criterion variable (i.e., general health). Moreover, each scale used in the current study showed adequate parameters for internal reliability, with values exceeding the minimum threshold of 0.65 (DeVellis, 2016).

**Table 1:** Mean, SD, and correlation among study variables.

			*R*						
	*M*	*SD*	1	2	3	4	5	6	7
(1) Gender^a^	0.47	0.50	*n.a.*	^∗^					
(2) Age	44.42	9.22	0.28^∗∗^	*n.a.*					
(3) Job tenure	14.65	8.96	0.05	0.61^∗∗∗^	*n.a.*				
(4) Transformational leadership	3.01	0.85	–0.04	0.03	0.07	(0.93)			
(5) Hardiness	3.13	0.38	0.02	0.08	0.01	0.18^∗∗^	(0.77)		
(6) Work engagement	4.29	1.09	0.01	0.09	0.13^∗^	0.27^∗∗^	0.52^∗∗∗^	(0.90)	
(7) General health	2.06	0.45	0.03	–0.03	0.04	0.20^∗∗∗^	0.14^∗^	0.33^∗∗∗^	(0.85)

*N = 358. ^∗^*p* < 0.05; ^∗∗^*p* < 0.01; ^∗∗∗^*p* < 0.001; ^*a*^1 = man; Cronbach’s alpha in brackets along the diagonal.*

### Model Testing

Table 2 displays the estimates of all the path coefficients, as well as the 95% bias-corrected bootstrapped Confidence Intervals (95% CI) concerning the indirect relationships included in the hypothesized model. With reference to the direct associations, transformational leadership showed a positive relationship with hardiness (*B* = 0.07, *SE* = 0.02, *p* < 0.01, 95% CI [0.02; 0.12]) and work engagement (*B* = 20, *SE* = 0.06, *p* < 0.01, 95% CI [0.08; 0.32]). Moreover, in the current sample a higher perception of transformational leadership had a positive association with employees’ general health status: *B* = 0.08, *SE* = 0.03, *p* < 0.01, 95% CI [0.02; 0.13]. This result supported *Hypothesis 1*.

**Table 2:** Path coefficients and indirect effects for mediation models.

	Path coefficients						Indirect effects		
	Hardiness (HR)		Work engagement (WE)		General health (GH)				
	*b*	*SE*	*b*	*SE*	*b*	*SE*			
Gender^a^	0.00	(0.04)	0.01	(0.11)	0.06	(0.05)			
Age	0.00	(0.00)	0.00	(0.01)	–0.01	(0.00)			
Job tenure	0.00	(0.00)	0.01^∗^	(0.01)	0.00	(0.00)			
Transformational leadership (TL)	0.07^∗∗^	(0.02)	0.20^∗∗^	(0.06)	0.08^∗∗^	(0.03)			
Hardiness (HR)			1.46^∗∗∗^	(0.14)	–0.06	(0.08)			
Work engagement (WE)					0.14^∗∗∗^	(0.03)			

							***b***	***SE***	**95% CI**

Total							0.04	(0.01)	0.02; 0.07
TL →HR → GH							–0.00	(0.01)	–0.01; 0.01
TL →WE → GH							0.03	(0.01)	0.01; 0.05
TL →HR →WE → GH							0.01	(0.01)	0.01; 0.03

*N = 358. ^∗^*p* < 0.05; ^∗∗^*p* < 0.01; ^∗∗∗^*p* < 0.001; ^*a*^1 = man; 95% CI = 95% confidence interval using the bootstrap bias corrected method using 5000 samples. TL, transformational leadership; HR, hardiness; WE, work engagement; GH, general health. Standard error in brackets.*

Concerning the indirect relationships, the obtained results indicated that employees’ hardiness did not significantly mediate the relationship between transformational leadership and general health, with *B* = -0.00, *SE* = 0.01, 95% CI [-0.01; 0.01]. Accordingly, *Hypothesis 2* was not supported. In contrast, work engagement acted as a significant mediator between the presence of a transformational leadership style enacted by the supervisor and the general health status reported by employees: *B* = 0.03, *SE* = 0.01, 95% CI [0.01; 0.05]. This result provided empirical support for *Hypothesis 3*.

The overall model tested on the current sample included transformational leadership (i.e., the independent variable), employees’ hardiness (included as first mediator), work engagement (included as second mediator), and general health (i.e., the model outcome).

Interestingly, results indicated that hardy personality and work engagement sequentially mediated the relationship between the perceived level of transformational leadership and employees’ general health. This evidence lent support to *Hypothesis 4*. In particular, transformational leadership was associated with higher levels of hardiness and work engagement, which in turn was related to a better perception of one’s general health status.

## Discussion

The current study aimed to investigate the role of transformational leadership within the motivational processes of the JD-R model and, more specifically, its role as an organizational resource related to greater levels of employees’ *hardiness*, which may enhance the degree of engagement experienced and, consequently, could be associated with better general health status. On the one hand, these findings are consistent with previous results suggesting that transformational leadership may have an indirect impact on work engagement, and the positive outcomes stemming from this positive motivational state toward work, through the reinforcement of personal resources (Mazzetti et al., 2016a). On the other hand, the current research represents one of the first attempts to explore the role of transformational leadership as an organizational resource related to higher levels of employees’ commitment, their feelings of control over daily tasks, and their tendency to perceive changes as challenges (i.e., hardiness). This result corroborates the definition of hardiness as a malleable personal characteristic, and therefore an individual resource that could be reinforced when employees are exposed to an organizational setting that provides stimuli that can encourage its development and maintenance (Sudom et al., 2014). This research corroborates the current understanding of transformational leadership as a job resource that could effectively strengthen not only individual characteristics (i.e., hardiness), but also foster a positive, fulfilling psychological state of motivation, involvement, and commitment toward one’s work (i.e., work engagement). Consistent with previous empirical findings, the obtained results allow us to conclude that a supervisor exhibiting a transformational leadership style – in particular, individualized consideration, inspirational motivation, and idealized influence – could boost employees’ well-being and general health status (Jacobs et al., 2013; Nielsen and Daniels, 2016).

Although the obtained results indicated a positive direct association between transformational leadership and hardiness, this personal resource did not emerge as a significant mediator within the indirect relationship of leadership and employees’ health. Actually, hardiness showed a non-significant relationship with participants’ perception of general health. Although this finding disagrees with previous results (Eschleman et al., 2010), the study adds to understanding of the role of hardiness as a personal resource fostered by transformational leadership and positively associated with work engagement which, in turn, may positively affect employees’ health status.

In particular, this research enriches present knowledge of the motivational processes postulated by the JD-R model (Schaufeli and Taris, 2014) and highlights the crucial role of supervisors adopting transformational behaviors to foster employees’ well-being through the subsequent mediation of followers’ hardiness and work engagement.

Overall, the current study contributes to ongoing research on the underlying mechanism that could explain the positive impact of transformational leadership on employees’ health and well-being (Arnold, 2017). To date, research investigating the opportunity to boost followers’ hardiness through their exposure to specific leadership behaviors has been limited to the military context. For instance, Bartone (2006) assessed the relationship between effective leaders’ behaviors and a greater level of hardiness among members of military units. The core assumption postulates that military leaders could act as principal role models within their unit who shape followers’ understanding of and reactions to stressful events and experience concerning military operations. The present study allowed us to take one step further through the exploration of this relationship beyond a work context that is unique in its key features, functioning, and aims, such as military units. Moreover, a large body of empirical research has focused on the positive impact of transformational leaders on employees’ commitment, motivation, and dedication to their work, and thus their level of engagement (Raja, 2012; Breevaart et al., 2014). On the one hand, the current research was to advance the understanding of the variables involved in this relationship through the investigation of a multiple – that is, serial – mediating mechanism, that allowed for the integration of the psychological processes and that may explain the link between the availability of specific job resources (i.e., transformational leadership) and healthy human resources (i.e., reporting higher levels of general health and well-being).

## Conclusion

As with any research in this field, the current study had some unavoidable limitations. First of all, our analysis was based on cross-sectional data, and this limitation prevents us from drawing undisputable conclusions about the causal relationship linking the variables under investigation. Further research should employ a longitudinal research design that would unequivocally prove that transformational leadership may impact sequentially on employees’ hardiness and work engagement, with consequent repercussions on their general health status. Moreover, the current study was based on the definition of hardiness as a personality characteristic resulting from the combination of the three interrelated dimensions of commitment, control, and challenge (Maddi and Kobasa, 1984). Although this conceptualization is widely supported in the existing literature, several studies have revealed that these three core dimensions are differently related to several indicators of work-related well-being (Ladstätter et al., 2018). Hence, future research avenues will need to investigate the mediating role of each facet of hardiness within the association between transformational leadership and employees’ level of engagement. A further limitation entails the nature of our sample, which consisted exclusively of white-collar workers employed in a single company, almost all of whom had a permanent employment contract. Thus, future research could test the current model in different working populations (e.g., blue-collar workers) and companies to corroborate these findings and test whether they are generalizable across different contexts. Moreover, given the recognized role of hardiness as a resilience resource, which enables employees to cognitively evaluate perceived stressors as being less harmful and unfavorable (Maddi, 2007), a significant contribution to the current literature on hardiness would derive from the exploration of its protective role among employees experiencing a condition that is traditionally recognized as one of the major job stressors, namely, job insecurity (Sverke et al., 2002). Thus, a valuable contribution could stem from a replication of the current model among temporary employees.

## Practical Implications

The current study provides evidence supporting the hypothesis that transformational leadership may trigger a motivational process by fostering employees’ hardiness, their level of work engagement, and, consequently, their health status. Although in the current study, hardiness did not show a significant relationship with employees’ health, the serial mediation model highlighted the mediating role of this personal characteristic in fostering the level of motivation and involvement toward one’s job (i.e., work engagement), which, in turn, may foster a general level of well-being among employees.

According to the present findings, the predictor of this virtuous cycle lies in the transformational leadership style adopted by the supervisors, and the literature has emphasized that transformational leadership behaviors can be learned and developed (Richter et al., 2016). Therefore, a decisive concrete indication stemming from the obtained results involves the opportunity to foster transformational leadership behaviors among supervisors, to subsequently enhance the levels of hardiness and engagement among employees who, in turn, experience better general health status.

More specifically, the implementation of training interventions aimed at enhancing transformational skills among supervisors should consider the interplay among personal, behavioral, and environmental factors in the leadership enhancement process. Empirical research has revealed that participants in transformational leadership development interventions were more prone to exhibit suitable behaviors when they experienced more positive psychological states (Mason et al., 2014). Therefore, with the purpose of activating a process that may result in better general health status, considering leaders’ psychological state would facilitate an effective change in their actual conduct.

On the whole, it can be concluded that organizations willing to rely on a healthy workforce should implement intervention strategies designed to train managers in practices and policies pertaining to a transformational leadership style, to boost employees’ hardy personality and, consequently, their level of engagement and health conditions.

## Author Contributions

GM, MV, and GP: conceptualization. GM and MV: formal analysis. GP and LP: investigation and writing – review and editing. GM: methodology. LP: supervision. MV and GP: writing – original draft.

## Conflict of Interest Statement

The authors declare that the research was conducted in the absence of any commercial or financial relationships that could be construed as a potential conflict of interest.
